# Upregulation of IL-4 receptor signaling pathway in circulating ILC2s from asthma patients

**DOI:** 10.1016/j.jacig.2022.07.007

**Published:** 2022-09-12

**Authors:** Rie Baba, Hiroki Kabata, Yoshitaka Shirasaki, Takashi Kamatani, Mai Yamagishi, Misato Irie, Risa Watanabe, Masako Matsusaka, Katsunori Masaki, Jun Miyata, Kazuyo Moro, Sotaro Uemura, Koichi Fukunaga

**Affiliations:** a Division of Pulmonary Medicine, Department of Medicine, Keio University School of Medicine, Tokyo, Japan; b Graduate School of Pharmaceutical Sciences, The University of Tokyo, Tokyo, Japan; c Laboratory for Medical Science Mathematics, Department of Biological Sciences, Graduate School of Science, The University of Tokyo, Tokyo, Japan; d Department of AI Technology Development, M&D Data Science Center, Tokyo Medical and Dental University, Tokyo, Japan; e Division of Precision Cancer Medicine, Tokyo Medical and Dental University Hospital, Tokyo, Japan; f Live Cell Diagnosis, Ltd, Asaka, Saitama, Japan; g Division of Infectious Diseases and Respiratory Medicine, Department of Internal Medicine, National Defense Medical College, Saitama, Japan; h Laboratory for Innate Immune Systems, RIKEN Center for Integrative Medical Sciences, Yokohama, Kanagawa, Japan; i Laboratory for Innate Immune Systems, Department of Microbiology and Immunology, Graduate School of Medicine, Osaka University, Suita, Osaka, Japan; j Laboratory for Innate Immune Systems, Immunology Frontier Research Center, Osaka University, Suita, Osaka, Japan; k Department of Biological Sciences, Graduate School of Science, The University of Tokyo, Tokyo, Japan

**Keywords:** Asthma, live cell imaging, biomarker, group 2 innate lymphoid cells

## Abstract

**Background:**

Group 2 innate lymphoid cells (ILC2s) produce type 2 cytokines by stimulation with epithelial cell–derived cytokines and are implicated in the pathogenesis of various allergic diseases, including asthma. However, differences in the molecular characteristics of ILC2s between patients with asthma and healthy subjects remain unclear.

**Objective:**

We sought to evaluate differences in cytokine production capacity and gene expression profile of ILC2s in the peripheral blood of patients with asthma and healthy subjects.

**Methods:**

We evaluated ILC2s derived from 15 patients with asthma and 7 healthy subjects using flow cytometry, live-cell imaging of secretion activity analysis, and RNA-sequencing.

**Results:**

ILC2s were sorted as CD45^+^Lineage^−^CRTH2^+^CD127^+^CD161^+^ cells from the peripheral blood of patients with asthma and healthy subjects, and the number of ILC2s was decreased in patients with asthma (851 ± 1134 vs 2679 ± 3009 cells/20 mL blood; *P* = .0066). However, patient-derived ILC2s were activated to produce more IL-5 and IL-13 in response to stimulation with IL-2, IL-33, and thymic stromal lymphopoietin compared with healthy subject–derived ILC2s (*P* = .0032 and *P* = .0085, respectively). Furthermore, RNA-sequencing analysis revealed that patient-derived ILC2s had different gene expression profiles, such as increased expression in cell growth–related genes (*CDKN1b*, *CCNG2*, *CCND2*, *CCN1*), prostaglandin E receptor (*PTGER2*), and IL-4 receptor. In addition, a gene set of the IL-4 receptor signaling pathway was significantly upregulated in ILC2s in patients with asthma (*P* = .042).

**Conclusions:**

Our results suggest that circulating ILC2s in patients with asthma are preactivated via the IL-4 receptor signaling pathway and produce IL-5 and IL-13 vigorously by stimulation.

## Introduction

The pathophysiology of asthma is characterized by chronic airway inflammation and variable airflow limitation, and various lymphoid cells, such as T_H_2 cells, group 2 innate lymphoid cells (ILC2s), and T_H_17 cells, are involved in the inflammation. Among them, ILC2s are directly activated by epithelial cell–derived cytokines, such as IL-33, IL-25, and thymic stromal lymphopoietin (TSLP), and produce large amounts of IL-5 and IL-13, which induce eosinophilic inflammation.^1^ Recent studies in humans and mice have shown that ILC2s are also associated with severe asthma by their capacity to become resistant to corticosteroids through TSLP and signal transducer and activator of transcription 5.^2^, ^3^, ^4^ Therefore, analyzing ILC2s in patients with asthma not only indicates the presence of an ILC2-mediated inflammation but may also serve as a biomarker for asthma severity and steroid resistance, which may be useful for personalized medicine.

Several research groups have assessed ILC2s in the peripheral blood of patients with asthma. However, the number of ILC2s is very low in the peripheral blood, ranging from 50 to 500 cells/mL, and there were conflicting results regarding whether or not the number of ILC2s was increased in the peripheral blood of patients with asthma.^5^, ^6^, ^7^, ^8^, ^9^, ^10^ Furthermore, the rarity of ILC2s makes it difficult to analyze cytokine production or gene expression profiles using conventional methods, such as ELISA and PCR. We previously developed the live-cell imaging of secretion activity (LCI-S) analysis system.^11^^,^^12^ This system is a microscope-based assay combining fluorescence immunoassay and near-field illumination by total internal reflection fluorescence microscopy,^13^ which is dedicated to the analysis of small numbers of cells, because cytokine production can be assessed at the single-cell level. Here, we applied this analysis system to evaluate the characteristics of ILC2s in the peripheral blood of patients with asthma and healthy subjects, revealing the distinct properties of circulating ILC2s in patients with asthma.

## Results and discussion

**Fig 1:**
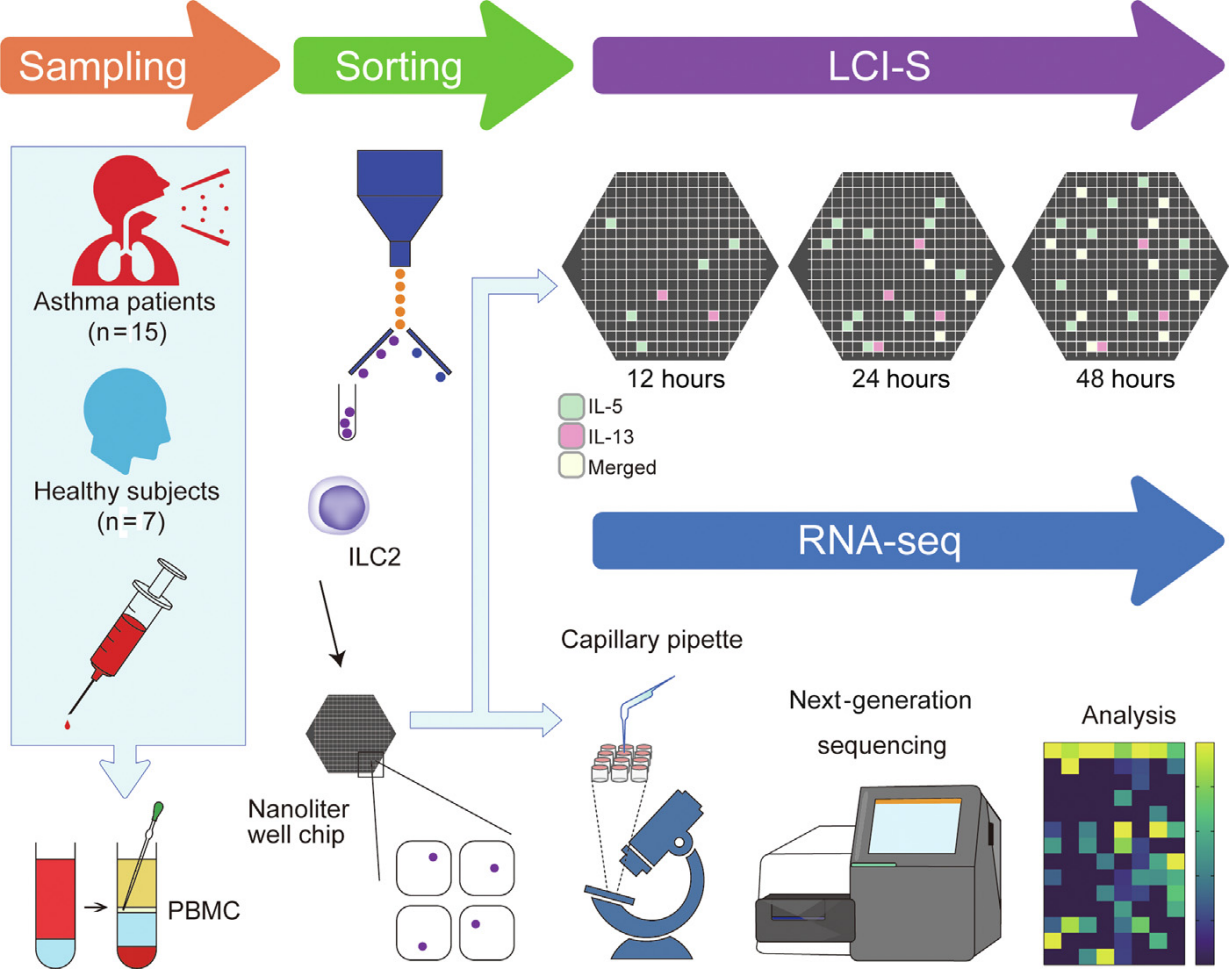
Cell analysis workflow. **A,** PBMCs were separated from 20 mL of peripheral blood. ILC2s were then sorted using a cell sorter. Sorted ILC2s were placed on a nanoliter-well array chip, and LCI-S analysis and RNA-seq analysis were performed separately.

To analyze the molecular characteristics of ILC2s, we used the LCI-S assay system combined with RNA-sequencing (RNA-seq) analysis^11^^,^^12^ (Fig 1). Briefly, PBMCs were separated from 20 mL of peripheral blood, and ILC2s were sorted as CD45^+^Lineage^−^CRTH2^+^CD127^+^CD161^+^ cells by flow cytometry (see Fig E1 in this article’s Online Repository at www.jaci-global.org). ILC2s were then placed on a nanoliter-well array chip consisting of 996 cubic wells with 80 μm per side. In each well of the chip, the capture antibodies were fixed on the bottom surface, and when cytokines were produced from ILC2s, they were fixed on the bottom and sandwiched by the fluorescence detection antibodies in the medium (see Fig E2 in this article’s Online Repository at www.jaci-global.org). The immunocomplexes on the bottom were illuminated by evanescent field excitation using total internal reflection fluorescence microscopy, resulting in continuous monitoring of the secretion activity of each cell (Fig E2). We stimulated ILC2s using IL-2 + IL-33 + TSLP and assessed IL-5 and IL-13 production for 48 hours. In addition, some ILC2s were collected individually from the nanoliter-well array chip with a dedicated capillary pipette before stimulation, and single-cell RNA-seq analysis was performed using SMART-Seq (Fig 1).

**Fig 2:**
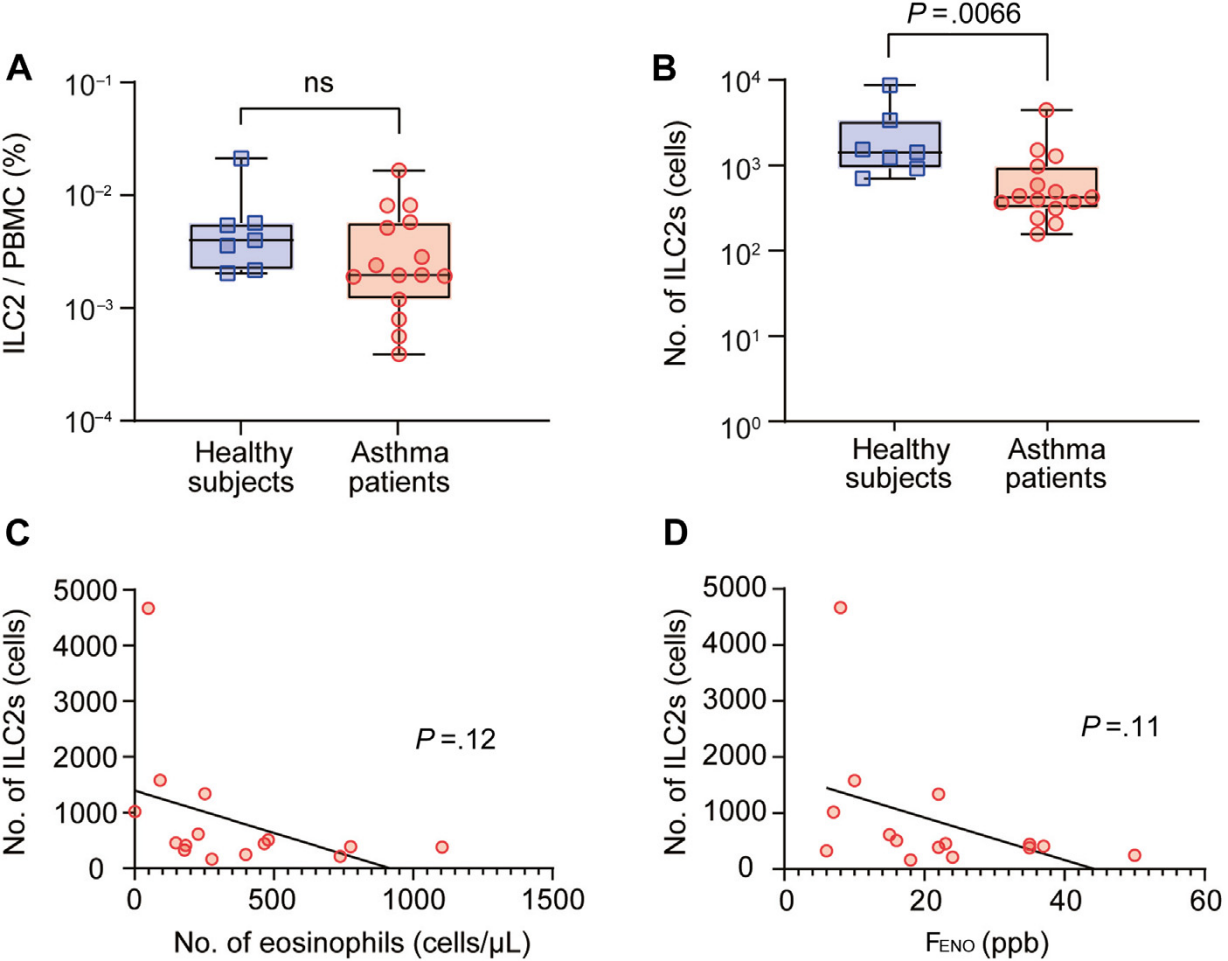
Differences in proportion and number of ILC2s. **A,** Proportion of ILC2s/PBMCs. **B,** Number of ILC2s in 20 mL of peripheral blood. **C** and **D,** Correlation between number of ILC2s with number of eosinophils in peripheral blood (Fig 2, *C*) and Feno (Fig 2, *D*). *F**eno*, Fractional exhaled nitric oxide; *ns*, not significant; *ppb*, parts per billion. Mean ± SEM. Mann-Whitney *U* test (Fig 2, *A* and *B*) and simple linear regression test (*C* and *D*).

We analyzed ILC2s in the peripheral blood of 15 patients with asthma compared with that of 7 healthy subjects (Table I). The patients with moderate to severe asthma who were treated with inhaled corticosteroids and other controllers (Global INitiative for Asthma treatment step 4 or 5) were enrolled, but patients receiving biologics or systemic corticosteroids were excluded. The phenotypes of the patients were diverse; 40% (6 patients) were eosinophil predominant (≥300 cells/μL), 27% (4 patients) had elevated fractional exhaled nitric oxide (≥30 ppb), and 47% (7 patients) were atopic. The absolute number of ILC2s was significantly reduced in patients with asthma compared with that in healthy subjects (851 ± 1134 vs 2679 ± 3009 cells/mL; *P* = .0066; Fig 2, *A* and *B*) although the decrease in the proportion (ILC2s/PBMC) was not statistically significant. It has been reported that allergen exposure and viral infection decreased ILC2 numbers in the peripheral blood and increased them in the lungs,^14^^,^^15^ and that the number of ILC2s did not correlate with the severity of asthma.^5^ Indeed, previous studies on the proportion or number of ILC2s in the peripheral blood of patients with asthma have shown conflicting results.^5^, ^6^, ^7^, ^8^, ^9^, ^10^ We further analyzed the relationship between the number of ILC2s and eosinophil counts or fractional exhaled nitric oxide levels, but found no correlation (Fig 2, *C* and *D*). Thus, these data suggest that the proportion or number of ILC2s in the peripheral blood is not a useful biomarker for asthma pathogenesis.

**Table I:** Characteristics of patients with asthma and healthy subjects in this study

Characteristic	Healthy subjects (n = 7)	Patients with asthma (n = 15)	*P* value
Demographic data			
Age (y), mean ± SD	47.1 ± 11.1	61.3 ± 16.6	NS∗
Sex: female/male, n (%)	4 (57.1)/3 (42.9)	8 (53.3)/7 (46.7)	NS†
Body mass index, mean ± SD	22.2 ± 2.7	24.6 ± 5.7	NS∗
Smoking history, n (%)	2 (28.5)	4 (26.7)	NS†
Comorbidities, n (%)			
Atopic dermatitis	0 (0)	3 (20)	NS†
Allergic rhinitis	1 (14.3)	2 (13.3)	NS†
Laboratory data			
Eosinophils (/μL), mean ± SD	NA	357.8 ± 309	
Total IgE (IU/mL), mean ± SD	62.7 ± 28.2	681.2 ± 1569	NS∗
FEV_1_ (%predicted), mean ± SD	NA	78.1 ± 27.6	
FEV_1_/FVC (%), mean ± SD	NA	67.5 ± 18.2	
Feno (ppb), mean ± SD	NA	21.9 ± 12.7	
Treatment			
ICS, n (%)		15 (100)	
Daily dose of ICS (μg/d), mean ± SD		822 ± 262	
LABA, n (%)		15 (100)	
LAMA, n (%)		3 (20)	
LTRA, n (%)		15 (100)	
GINA step 4/5, n (%)		1 (6.7)/14 (93.3)	

*F**eno*, fractional exhaled nitric oxide; *FVC*, forced vital capacity; *GINA*, Global INitiative for Asthma; *ICS*, inhaled corticosteroid; *LABA*, long-acting β-agonist

*LAMA*, long-acting muscarinic antagonist; *LTRA*, leukotriene receptor antagonist; *NA*, not applicable/available; *NS*, not significant; *ppb*, parts per billion.

∗ Mann-Whitney *U* test.

† Fisher exact test.

**Fig 3:**
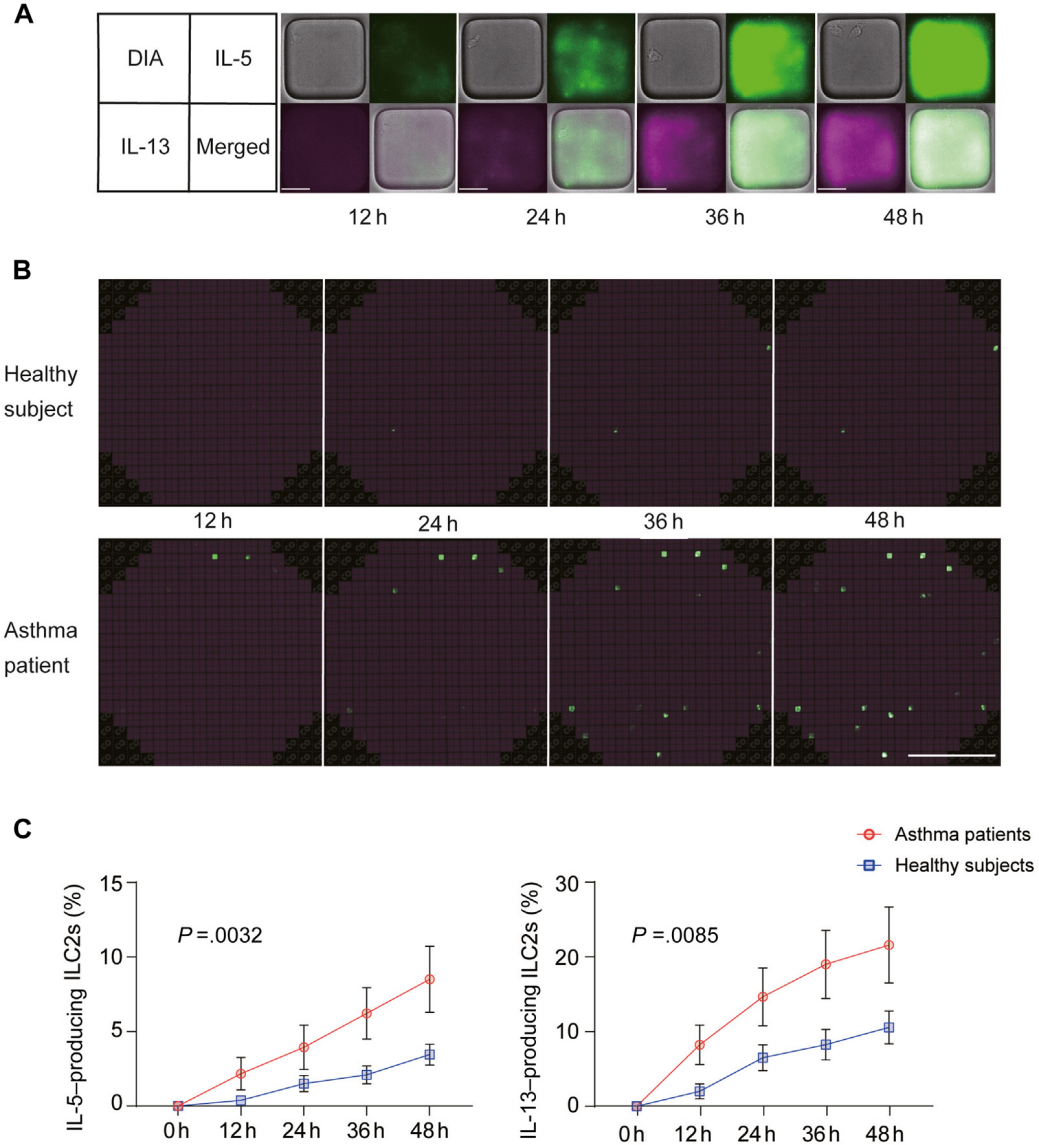
LCI-S analysis of ILC2s. **A** and **B,** Representative data of a single well (Fig 3, *A*) and overview (Fig 3, *B*) of nanoliter chip during LCI-S analysis. ILC2s produced IL-5 and IL-13 under the stimulation of IL-2 + IL-33 + TSLP. **C,** Cumulative data of the percentages of wells containing ILC2s producing IL-5 or IL-13 above quantification limits (>10σ). *DIA*, Diascopic illumination. Mean ± SEM. Two-way repeated-measure ANOVA.

Next, we performed LCI-S analysis to evaluate the differences in cytokine production capacity of ILC2s. In a nanoliter-well array chip, a deep learning algorithm was used to detect wells containing ILC2s,^16^ and the fluorescence intensity of each well was measured. Upon IL-2 + IL-33 + TSLP stimulation, ILC2s moved around in the wells and gradually produced IL-5 and IL-13 (see Movie E1 in this article’s Online Repository at www.jaci-global.org; Fig 3, *A*). The number of wells containing IL-5– or IL-13–producing ILC2s increased over time and was higher in patients with asthma (see Movie E2 in this article’s Online Repository at www.jaci-global.org; Fig 3, *B*). When we calculated the percentage of wells containing ILC2s that produced IL-5 or IL-13 above the levels of the detection limit, both were significantly higher in patients with asthma than in healthy subjects (*P* = .0032 and *P* = .0085, respectively; Fig 3, *C*), indicating that ILC2s in the peripheral blood of patients with asthma produced more cytokines in response to stimulation. In recent years, other researchers have also suggested that the production of cytokines from ILC2s in patients with asthma is higher than that in healthy patients,^6^ even though the number of ILC2s is not increased.^10^ However, the previous studies required long-term (7 days) culture methods or nonphysiological stimulation (phorbol 12-myristate 13-acetate and ionomycin). Such long-term culture or phorbol 12-myristate 13-acetate and ionomycin may forcefully activate ILC2s, which are not activated under physiological conditions. Because the LCI-S analysis can detect subtle amounts of cytokines at the single-cell level, it is possible to detect the difference in cytokine production from ILC2s between patients with asthma and healthy subjects within 48 hours of measurement under cytokine stimulation.

**Fig 4:**
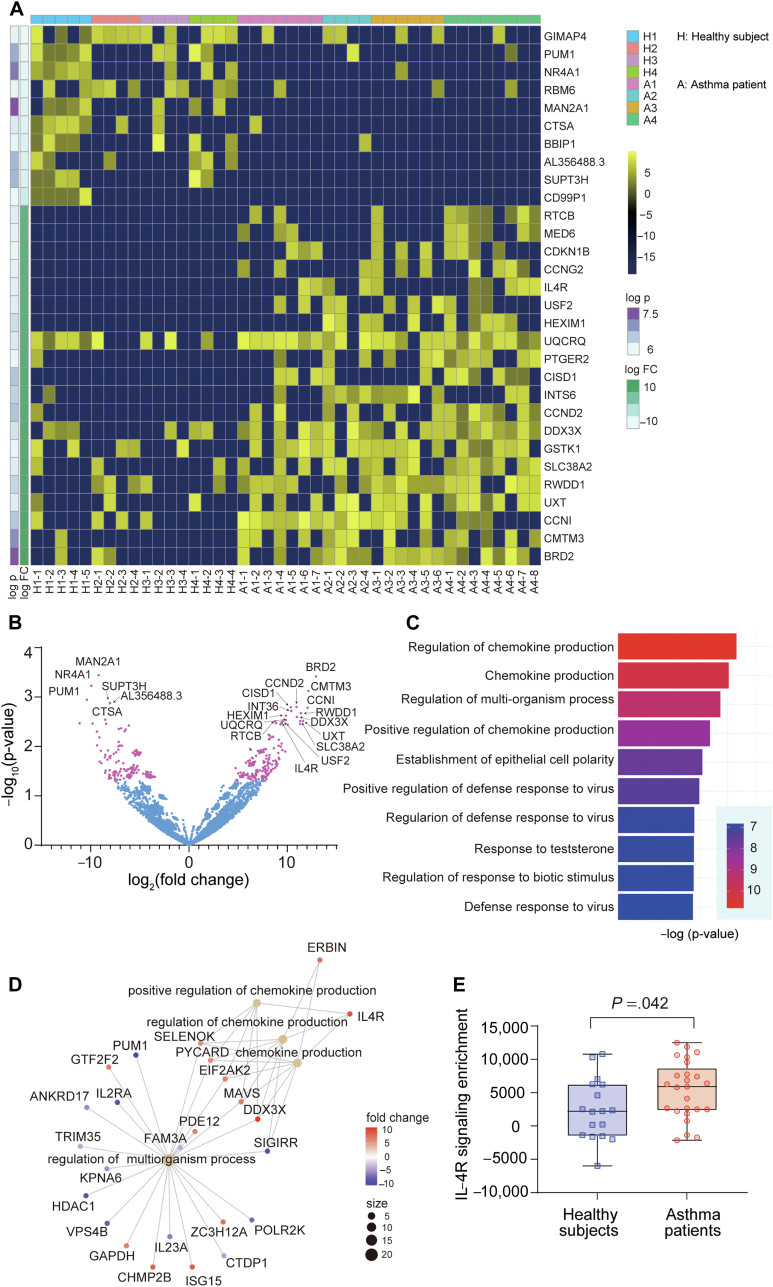
Differences in gene expression profiles of ILC2s. **A,** Heatmap of the top 30 DEGs between 27 ILC2s from patients with asthma and 17 ILC2s from healthy subjects. **B,** Volcano plot of DEGs. **C,** Gene ontology analysis of DEGs. **D,** Cnetplot of gene ontology analysis. **E,** Single sample gene set enrichment analysis for IL-4 receptor signaling pathway. Mann-Whitney *U* test. *DEG*, Differentially expressed gene; *FC*, fold change.

Finally, to clarify the molecular mechanism of the different cytokine producing capacities of ILC2s in patients with asthma, we performed single-cell RNA-seq analysis on 44 ILC2s randomly selected from 4 patients with asthma and 4 healthy subjects before stimulation. We identified significantly different gene expressions between them; cell growth–related genes (*CDKN1b*, *CCNG2*, *CCND2*, and *CCN1*), prostaglandin E receptor (*PTGER2*), and IL-4 receptor (*IL4R*) were significantly upregulated in ILC2s derived from patients with asthma (Fig 4, *A* and *B*). Gene ontology analysis with a cnetplot revealed that some gene sets, such as chemokine production and multiorganism process, were enriched in patient-derived ILC2s (Fig 4, *C* and *D*). It has been reported that IL-4, mainly produced by T_H_2 cells and basophils, enhances activation of ILC2s to produce IL-5 and IL-13.^17^ As shown previously, the expression of *IL4R* was significantly upregulated in ILC2s in patients with asthma (*P* = .042; log fold change = 217.13), and *IL4R* affects all gene ontologies associated with chemokine production. Furthermore, we confirmed that *IL4R* (CD124) expression was significantly increased in patients with asthma compared with that in healthy subjects by flow cytometry (see Fig E3 in this article’s Online Repository at www.jaci-global.org). To validate whether the IL-4 receptor signaling pathway was primed in ILC2s of patients with asthma, we performed single-sample gene set enrichment analysis^18^ and revealed a significant enrichment of IL-4 receptor signaling pathway genes (*AKT1*, *GRB2*, *IL2RG*, *IL4*, *IL4R*, *IRS1*, *JAK1*, *JAK3*, *RPS6KB1*, *SHC1*, and *STAT6*) in ILC2s of patients with asthma (*P* = .042; Fig 4, *E*). Thus, these data suggest that ILC2s in patients with asthma are preactivated via the IL-4 receptor signaling pathway, which may be a cause of production of more type 2 cytokines in response to the stimulation.

In summary, we revealed the molecular characteristics of ILC2s in the peripheral blood of patients with asthma; the number of ILC2s in the patients was decreased, but the patient-derived ILC2s were highly responsive to the cytokine stimulation and produced high amounts of IL-5 and IL-13. Furthermore, as the underlying mechanism for the activated phenotype, we identified that the IL-4 receptor signaling pathway was primed in the patient-derived ILC2s. A recent study has shown that the number and cytokine expression of ILC2s are significantly decreased in patients with asthma receiving anti–IL-4 receptor antibodies.^19^ Therefore, anti–IL-4 receptor antibodies may be effective in the patients with preactivated ILC2s via enhanced IL-4 receptor signaling pathway. As a limitation of this study, although patients with asthma have various phenotypes, this pilot study included a limited number of patients, and a detailed phenotype-specific analysis was not performed. Further studies are warranted to evaluate the correlation between the molecular characteristics of ILC2s and the disease phenotype or response to biologics.

Finally, we revealed the molecular characteristics of ILC2s in patients with asthma, which will contribute to personalized medicine and targeted therapy for asthma based on the phenotype of the lymphoid cells.

**Key messages**

• In patients with asthma, the number of ILC2s in peripheral blood was not increased, but the ability to produce type 2 cytokines was enhanced.
• Gene expression profiles of ILC2s were different between patients with asthma and healthy subjects, and a gene set of the IL-4 receptor signaling pathway was significantly upregulated.

## Supplementary data

Movie E1

Movie E2

Supplementary Material

Figure E1

Figure E2

Figure E3

## References

[1] Kabata H., Moro K., Koyasu S. (2018). The group 2 innate lymphoid cell (ILC2) regulatory network and its underlying mechanisms. Immunol Rev.

[2] Kabata H., Moro K., Fukunaga K., Suzuki Y., Miyata J., Masaki K. (2013). Thymic stromal lymphopoietin induces corticosteroid resistance in natural helper cells during airway inflammation. Nat Commun.

[3] Shikotra A., Choy D.F., Ohri C.M., Doran E., Butler C., Hargadon B. (2012). Increased expression of immunoreactive thymic stromal lymphopoietin in patients with severe asthma. J Allergy Clin Immunol.

[4] Liu S., Verma M., Michalec L., Liu W., Sripada A., Rollins D. (2018). Steroid resistance of airway type 2 innate lymphoid cells from patients with severe asthma: the role of thymic stromal lymphopoietin. J Allergy Clin Immunol.

[5] Yu Q.-N., Tan W.P., Fan X.-L., Guo Y.B., Qin Z.-L., Li C.-L. (2018). Increased group 2 innate lymphoid cells are correlated with eosinophilic granulocytes in patients with allergic airway inflammation. Int Arch Allergy Immunol.

[6] Jia Y., Fang X., Zhu X., Bai C., Zhu L., Jin M. (2016). IL-13 + type 2 innate lymphoid cells correlate with asthma control status and treatment response. Am J Respir Cell Mol Biol.

[7] Smit S.G., Chen R., Kjarsgaard M., Huang C., Oliveria J.-P., O’Byrne P.M. (2016). Increased numbers of activated group 2 innate lymphoid cells in the airways of patients with severe asthma and persistent airway eosinophilia. J Allergy Clin Immunol.

[8] Liu T., Wu J., Zhao J., Wang J., Zhang Y., Liu L. (2015). Type 2 innate lymphoid cells: a novel biomarker of eosinophilic airway inflammation in patients with mild to moderate asthma. Respir Med.

[9] Bartemes K.R., Kephart G.M., Fox S.J., Kita H. (2014). Enhanced innate type 2 immune response in peripheral blood from patients with asthma. J Allergy Clin Immunol.

[10] Drake L.Y., Bartemes K.R., Bachma K.A., Hagan J.B., Kita H. (2021). In vitro culture with cytokines provides a tool to assess the effector functions of ilc2s in peripheral blood in asthma. J Asthma Allergy.

[11] Shirasaki Y., Yamagishi M., Suzuki N., Izawa K., Nakahara A., Mizuno J. (2014). Real-time single-cell imaging of protein secretion. Sci Rep.

[12] Yamagishi M., Shirasaki Y. (2021). Live-cell imaging technique to visualize DAMPs release during regulated cell death. Methods Mol Biol.

[13] Liu T., Yamaguchi Y., Shirasaki Y., Shikada K., Yamagishi M., Hoshino K. (2014). Single-cell imaging of caspase-1 dynamics reveals an all-or-none inflammasome signaling response. Cell Rep.

[14] Chen R., Smith S.G., Salter B., El-Gammal A., Oliveria J.-P., Obminski C. (2017). Allergen-induced increases in sputum levels of group 2 innate lymphoid cells in subjects with asthma. Am J Respir Crit Care Med.

[15] Winkler C., Hochdörfer T., Israelsson E., Hasselberg A., Cavallin A., Thörn K. (2019). Activation of group 2 innate lymphoid cells after allergen challenge in asthmatic patients. J Allergy Clin Immunol.

[16] Kamatani T., Fukunaga K., Miyata K., Shirasaki Y., Tanaka J., Baba R. (2017). Construction of a system using a deep learning algorithm to count cell numbers in nanoliter wells for viable single-cell experiments. Sci Rep.

[17] Motomura Y., Morita H., Moro K., Nakae S., Artis D., Endo T.A. (2014). Basophil-derived interleukin-4 controls the function of natural helper cells, a member of ILC2s, in lung inflammation. Immunity.

[18] Barbie D.A., Tamayo P., Boehm J.S., Kim S.Y., Moody S.E., Dunn I.F. (2009). Systematic RNA interference reveals that oncogenic KRAS-driven cancers require TBK1. Nature.

[19] Patel G., Pan J., Ye L., Shen X., Rosloff D., D’Souza S.S. (2020). Blockade of IL-4Rα inhibits group 2 innate lymphoid cell responses in asthma patients. Clin Exp Allergy.

