# Supplementary material for: Upregulation of IL-4 receptor signaling pathway in circulating ILC2s from asthma patients
Source: J Allergy Clin Immunol Glob. 2022 Sep 12;1(4):299–304. doi: 10.1016/j.jacig.2022.07.007 (PMC10509846; doi:10.1016/j.jacig.2022.07.007)
Supplement: Supplementary Material [file mmc3.docx]

**Online Repository**

**Methods**

**Study design**

We enrolled Japanese patients with asthma (age ≥ 20 years) who were diagnosed and treated by a respiratory specialist at the Keio University Hospital. Patients treated with biologics or oral corticosteroids and patients with widespread lung disease or uncontrolled malignant tumors were excluded. Patient demographics and clinical characteristics, biomarker levels, and pulmonary function were assessed under stable treatment at the time of enrollment. Serum total IgE levels were measured using a fluorescence enzyme immunoassay (Mitsubishi Chemical Medience Corporation, Tokyo, Japan). Pulmonary function was assessed using the CHESTAC-9800 spirometer (CHEST, Tokyo, Japan). Predicted forced expiratory volume in 1 second (FEV_1_) was calculated using the lambda-mu-sigma method recommended for the Japanese population by the Japanese Respiratory Society. Data on asthma symptoms and current asthma treatment were captured using a self-completed questionnaire and medical records, respectively. The study protocol was approved by the Institutional Review Board (IRB) of the Keio University School of Medicine (IRB approval number: 20090009). This study was conducted in compliance with the Declaration of Helsinki. All patients provided written informed consent, and patient anonymity was preserved using methods approved by the IRB.

**Reagents**

For cell sorting of ILC2s, we used FITC-conjugated lineage antibody cocktails of human CD3 (UCHT1), CD14 (HCD14), CD16 (3G8), CD19 (HIB19), CD20 (2H7), CD56 (HCD56), PE-conjugated anti-human CD127 (A019D5), PE/Cy7-conjugated anti-human CD161 (HP-3G10) antibodies (BioLegend, San Diego, CA, USA), and Alexa Fluor 647-conjugated anti-human CRTH2 (BM16) antibody (BD Biosciences, San Jose, CA, USA). For analysis of CD124 expression, we used BV421-conjugated anti-human CD124 (G077F6) antibodies and isotype control antibodies (BioLegend, San Diego, CA, USA). For the LCI-S system, we used anti-human IL-13 and anti-human IL-5 antibodies (R&D Systems, Minneapolis, MN, USA) and recombinant human IL-2, IL-33, and TSLP (R&D Systems, Minneapolis, MN, USA).

**Isolation of human ILC2s**

Peripheral blood was obtained from patients and healthy volunteers at Keio University hospital. Mononuclear cells were obtained using Lymphoprep™ (Axis-Shield, Dundee, UK), according to the manufacturer’s protocols. CD45^+^Lineage^-^CRTH2^+^CD127^+^CD161^+^PI^-^ cells were sorted using a MoFlo™ XDP flow cytometer (Beckman Coulter, Brea, CA, USA).

**LCI-S analysis**

LCI-S analysis was performed as reported previously^E1,E2^. Briefly, a fully automated inverted microscope (ECLIPSE Ti2-E; Nikon, Tokyo, Japan) was used for time-lapse imaging using total internal reflection fluorescence (TIRF) illumination of LED light (X-Cite XLED1, mounted with RLX: 615 -655 nm, Excelitas Technologies Corp., Waltham, MA) through white-light TIRF optics (high-performance Epi-fl illuminator module TI-SFL). The optical configurations used were the following: excitation filter = FF01-635/18; emission filter = FF01-692/40; and dichroic mirror = FF560/659-Di01. These optical filters were purchased from Semrock (Rochester, NY, USA). We introduced sorted ILC2s into a nanoliter-well array chip, which comprised a polydimethylsiloxane, amorphous fluorocarbon polymer CYTOP™, and glass with immobilized capture antibodies. After introducing ILC2s into the chip, detection medium containing Cy3- or CF660R-labeled detection antibodies and cytokine stimuli (200 U/mL IL-2, 1 μg/mL IL-33, and 1 μg/mL TSLP) were applied, and observation was started immediately. Bright-field and TIRF images of all nanoliter wells were acquired at 1-hour intervals for 60 hours. The bright-field images were used to identify and count the cells in each well and the TIRF images were used to measure the average fluorescence intensity of each well for estimation of cytokine production. These images were analyzed using commercially available software (NIS Elements 5.2, Nikon). Spatial inhomogeneity and temporal instability of the excitation light intensity were corrected for using the fluorescence signal of the cell-free wells (hereafter referred to as the background signal). The threshold for the identification of cytokine-producing cells was calculated from 1.4826 times the median absolute value of the background signal, which corresponds to the standard deviation (σ) of the background signal assuming a normal distribution. Here, 10 times σ was used as the limit of quantification (LOQ, 10σ).

**Cell recovery**

Single ILC2 collection was performed as reported previously^E1,E2^. Briefly, single cells were recovered using a glass capillary (L-Tip 15 µm 60° 15 mm, Yodaka Co., Ltd., Kawasaki, Japan) and a pneumatic microinjector (IM-11-2, NARISHIGE, Tokyo, Japan). The glass capillary was positioned using a micromanipulator (Quick Pro, Micro Support Co. Ltd., Shizuoka, Japan). The aspirated cell was ejected to 2 μL RNase-free water (06442-95, Nacalai tesque, Kyoto, Japan) for RNA-seq in a PCR tube. In this pilot study, collection of ILC2s from all subjects was not feasible owing to equipment and technical limitations. Therefore, we collected at least 4 ILC2s per subject from 4 patients and 4 healthy subjects, who were randomly selected. Recovered cells were immediately frozen in liquid nitrogen and stored at -80°C until gene expression analysis.

**RNA sequencing**

We synthesized cDNA libraries using SMART-Seq v4 3’-DE Kits (Takara Bio Inc., Shiga, Japan) according to the manufacturer’s instructions, with some modifications, adding synthetic oligo RNA to suppress undesirable concatemers. A total of 3,892 copies of ERCC spike-in RNA were added to each sample. The cDNA was purified using Agencourt AMPure XP magnetic beads (Beckman Coulter). Library quality check was performed using an Agilent 2100 Bioanalyzer (Agilent Technologies, Santa Clara, CA, USA) and Agilent High Sensitivity DNA Kits (5067-4626). Degraded or low-yield samples were removed. Qubit High Sensitivity assays (Thermo Fisher Scientific) were performed to quantify the cDNA in each library, and 5 or 6 libraries with different indexed primers were evenly pooled. Pooled cDNA (400 pg) was tagmented using Nextera XT DNA Library Prep Kits (Illumina, San Diego, CA, USA), as described in the protocol. Library size and cDNA amount were quantified using an Agilent High Sensitivity DNA Kit and Qubit High Sensitivity assays, respectively. Pooled libraries were sequenced using 91-bp paired-end sequencing on a MiSeq instrument (Illumina). After library demultiplexing and adaptor trimming, we aligned read 1 to reference sequences (human: GRCh38.90) using TopHat, and the read counts were calculated using HTSeq.

**Normalization of count data**

In RNA-seq from small numbers of cells, stochastic dropout events that occur with amplification of low copy number transcripts are more frequent. Counts per million (CPM), which is a correction method based on total counts, exhibited a resource-dependent cell-to-cell bias, particularly in RNA-seq data on single ILC2s. Therefore, in the analysis of per-cell data, we applied a modified CPM in which the total counts of genes whose expression levels were in the top 300 in each cell were used as the population counts. Then, CPM+1 values were log2 transformed and genes with mean log2 (CPM+1) values were over three were used for the following analysis.

**Differential expression analysis**

Differential expression analysis was performed using the ‘limma’ R package (v3.42.2)^E3^. Gene ontology and cnetplot were analyzed using the “enrichplot” package in R (v1.13.1). Duplicate Correlation function was used to estimate correlations between biological replicates in the lmFit linear modelling step with a random effect. Genes with a p-value <0.05 and | a log fold-change (logFC) | over 1.5 were selected as differentially expressed genes. Additionally, to build a more complete picture of the IL-4 signaling enrichment, we performed ssGSEA with BIOCARTA_IL4_PATHWAY (https://www.gsea-msigdb.org/gsea/msigdb/cards/BIOCARTA_IL4_PATHWAY) using expression data^E4^.

**Statistical analyses**

Categorical variables were summarized as the number and percentage of patients, and continuous variables were summarized as mean and standard deviation (SD). Statistical differences were assessed using the Mann-Whitney U test with a 2-sided level of significance of 0.05. All statistical analyses were performed using SAS version 9.4 (SAS Institute Inc., Cary, NC, USA), JMP version 14 (SAS Institute Inc., Cary, NC, USA), and R version 3.6.3.

**Online References**

E1. [Shirasaki](https://pubmed.ncbi.nlm.nih.gov/?sort=date&term=Shirasaki+Y&cauthor_id=24751898) Y, [Yamagishi](https://pubmed.ncbi.nlm.nih.gov/?sort=date&term=Yamagishi+M&cauthor_id=24751898) M, [Suzuki](https://pubmed.ncbi.nlm.nih.gov/?sort=date&term=Suzuki+N&cauthor_id=24751898) N, [Izawa](https://pubmed.ncbi.nlm.nih.gov/?sort=date&term=Izawa+K&cauthor_id=24751898) K, [Nakahara](https://pubmed.ncbi.nlm.nih.gov/?sort=date&term=Nakahara+A&cauthor_id=24751898) A, [Mizuno](https://pubmed.ncbi.nlm.nih.gov/?sort=date&term=Mizuno+J&cauthor_id=24751898) J, et al. Real-time single-cell imaging of protein secretion. Sci Rep. 2014;4:4736. https://doi.org/10.1038/srep04736.

E2. [Yamagishi](https://pubmed.ncbi.nlm.nih.gov/?sort=date&term=Yamagishi+M&cauthor_id=34050484) M, [Shirasaki](https://pubmed.ncbi.nlm.nih.gov/?sort=date&term=Shirasaki+Y&cauthor_id=34050484) Y. Live-cell imaging technique to visualize DAMPs release during regulated cell death. Methods Mol Biol. 2021;2274:337-52. https://doi.org/10.1007/978-1-0716-1258-3_28.

E3. [Ritchie](https://pubmed.ncbi.nlm.nih.gov/?sort=date&term=Ritchie+ME&cauthor_id=25605792) ME, [Phipson](https://pubmed.ncbi.nlm.nih.gov/?sort=date&term=Phipson+B&cauthor_id=25605792) B, [Wu](https://pubmed.ncbi.nlm.nih.gov/?sort=date&term=Wu+D&cauthor_id=25605792) D, [Hu](https://pubmed.ncbi.nlm.nih.gov/?sort=date&term=Hu+Y&cauthor_id=25605792) Y, [Law](https://pubmed.ncbi.nlm.nih.gov/?sort=date&term=Law+CW&cauthor_id=25605792) CW, [Shi](https://pubmed.ncbi.nlm.nih.gov/?sort=date&term=Shi+W&cauthor_id=25605792) W, et al. limma powers differential expression analyses for RNA-sequencing and microarray studies. Nucleic Acids Res. 2015;43:e47. https://doi.org/10.1093/nar/gkv007.

E4. [Barbie](https://pubmed.ncbi.nlm.nih.gov/?sort=date&term=Barbie+DA&cauthor_id=19847166) DA, [Tamayo](https://pubmed.ncbi.nlm.nih.gov/?sort=date&term=Tamayo+P&cauthor_id=19847166) P, [Boehm](https://pubmed.ncbi.nlm.nih.gov/?sort=date&term=Boehm+JS&cauthor_id=19847166) JS, [Kim](https://pubmed.ncbi.nlm.nih.gov/?sort=date&term=Kim+SY&cauthor_id=19847166) SY, [Moody](https://pubmed.ncbi.nlm.nih.gov/?sort=date&term=Moody+SE&cauthor_id=19847166) SE, [Dunn](https://pubmed.ncbi.nlm.nih.gov/?sort=date&term=Dunn+IF&cauthor_id=19847166) IF, et al. Systematic RNA interference reveals that oncogenic KRAS-driven cancers require TBK1. Nature. 2009;5;462:108-12. doi: 10.1038/nature08460.

**Supplementary Movie**

Movie 1. Representative high magnification imaging data of LCI-S analysis of ILC2 derived from an asthma patient.

Movie 2. Representative low magnification imaging data of LCI-S analysis of ILC2s in an asthma patient and a healthy subject.

**Supplementary Figure**

Figure E1. Gating strategy for sorting ILC2s.

Figure E2. Cytokine detection mechanism of LCI-S analysis before and after ILC2s produced cytokines.

Figure E3. *IL4R* (CD124) expression of ILC2s in asthma patients and healthy subjects. (A) Representative flow cytometry plot of CD124 expression of ILC2s in healthy subjects and asthma patients. (B) Mean fluorescence intensity (MFI) of CD124 expression (5 asthma patients vs 5 healthy subjects).
