# Supplementary figures and images for: Upregulation of IL-4 receptor signaling pathway in circulating ILC2s from asthma patients
Source: J Allergy Clin Immunol Glob. 2022 Sep 12;1(4):299–304. doi: 10.1016/j.jacig.2022.07.007 (PMC10509846; doi:10.1016/j.jacig.2022.07.007)

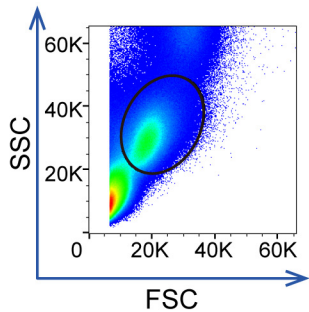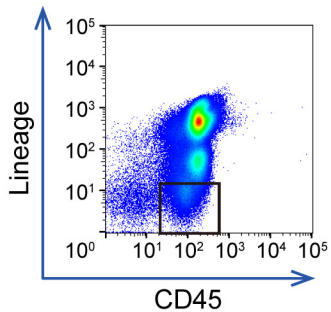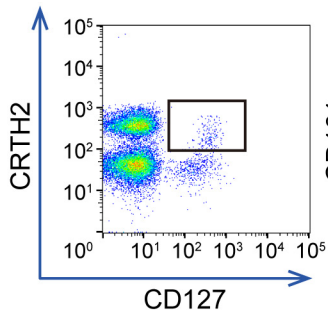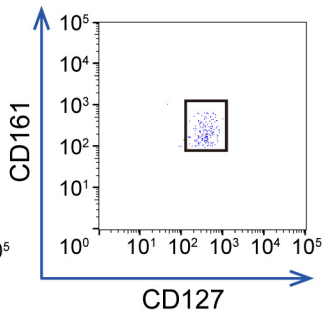

Supplement: Figure E1 [file mmc4.pdf]

A

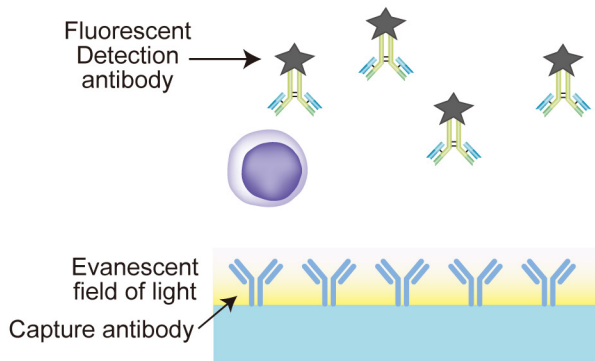

B

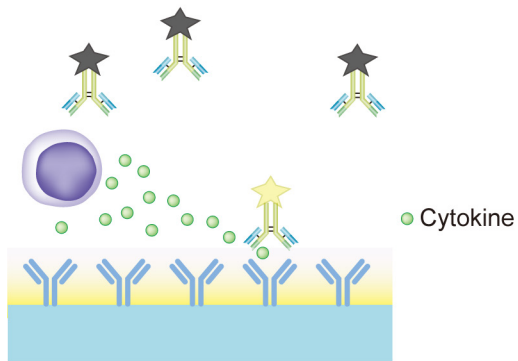

Supplement: Figure E2 [file mmc5.pdf]

A

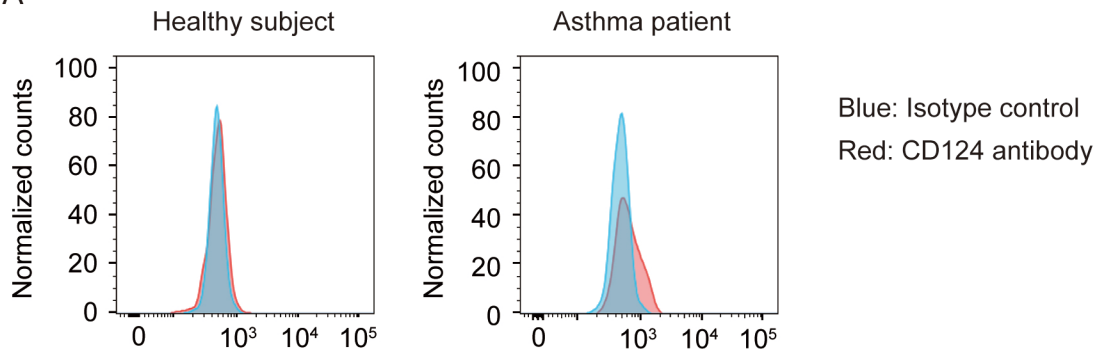

B

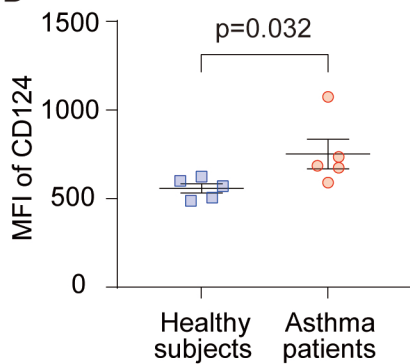

Supplement: Figure E3 [file mmc6.pdf]
